# Alteration of Collagen Content and Macrophage Distribution in White Adipose Tissue under the Influence of Maternal and Postnatal Diet in Male Rat Offspring

**DOI:** 10.3390/medicina59050888

**Published:** 2023-05-05

**Authors:** Robert Mujkić, Darija Šnajder Mujkić, Nenad Čekić, Ivana Ilić, Anđela Grgić, Željka Perić Kačarević, Valerija Blažićević

**Affiliations:** 1 Department of Anatomy, Histology, Embryology, Pathological Anatomy and Pathological Histology, Faculty of Dental Medicine and Health, Josip Juraj Strossmayer University of Osijek, 31000 Osijek, Croatia; rmujkic@fdmz.hr (R.M.);; 2 Department of Anatomy and Neuroscience, Faculty of Medicine, Josip Juraj Strossmayer University of Osijek, 31000 Osijek, Croatia; 3 Clinical Institute of Nuclear Medicine and Radiation Protection, University Hospital Osijek, 31000 Osijek, Croatia

**Keywords:** obesity, high-fat diet, collagen, inflammation, macrophages, adipose tissue

## Abstract

*Background and Objectives:* The extracellular matrix is important for adipose tissue growth, and numerous interactions between adipocytes and extracellular matrix components occur during adipose tissue development. The main objective of this study was to investigate the interaction and influence of maternal and postnatal diet on adipose tissue remodeling in Sprague Dawley offspring. *Materials and Methods:* 10 Sprague Dawley females were randomly divided into two groups at nine weeks of age and fed a standard laboratory diet or high-fat diet for six weeks. Then, they were mated, and after birth, their male rat offspring were divided into four subgroups according to diet. After euthanizing the offspring at 22 weeks of age, samples of subcutaneous, perirenal and epididymal adipose tissue were collected. Sections were stained with Mallory’s trichrome and analyzed by immunohistochemistry for CD68^+^ and CD163^+^ cells. *Results:* Staining of extracellular components showed higher collagen deposition in the perirenal and epididymal depot of offspring fed a high-fat diet. The number of CD163/CD68^+^ cells in the perirenal adipose tissue was lower in the CD-HFD group compared with other groups, and in the subcutaneous fat pad when the groups with modified diet were compared with those on non-modified diet. *Conclusion:* Morphological changes in adipose tissue, increased collagen deposition, and changes in macrophage polarization may be related to intergenerational changes in diet.

## 1. Introduction

Chronic inflammation has been linked to obesity in many different rodent models, with elevated TNF-α expression being shown in the adipose tissue (AT) of obese animals, which can be a mediator of insulin resistance (IR) [1,2]. Offspring in several rat models with high-fat diet (HFD)-fed mothers experienced a metabolic syndrome-like state [3]. Many processes that are physiologically important for normal AT function during remodeling can be studied in animal models. One of the advantages of studying AT in rodent models is that some processes related to AT physiology can occur in a very short time. For example, a 24-h fasting diet in mice leads to the loss of AT and the onset of remodeling processes with macrophage infiltration. After a 48-h HFD, an increase in adipocytes is already observed [4].

AT is the primary site of obesity-induced inflammation [5], it develops after three weeks of HFD, and hyperinsulinemia becomes apparent after 16 weeks [6]. Several other inflammatory indicators, including IL-1β, IL-6 and C-reactive protein (CRP) are also associated with obesity [7]. Pro-inflammatory cytokines such as TNF-α can be produced by many cell types but are predominately synthesized in stromal vascular fraction cells such as macrophages [8]. The subacute inflammatory state associated with obesity is believed to be induced and sustained by pro-inflammatory and anti-inflammatory cytokines, such as TNF-α, IL-6, and resistin. Chemokines, including monocyte chemoattractant protein 1, are crucial for the recruitment of macrophages to adipose tissue. The activation of intracellular pathways by these cytokines and chemokines promotes the development of insulin resistance and type 2 diabetes [9].

Besides the cytokine-induced inflammatory signaling pathways, in obesity, there is an infiltration of AT with immune cells, ranging with macrophage percentage from under 10% in lean mice and humans to 40–50% in obese mice and humans [5,10].

Although adipocytes have a significant impact on local changes that occur in the microenvironment, there is substantial evidence to suggest that macrophages also play a crucial role in remodeling processes. The functions and actions of the adipose tissue macrophages (ATMs) that reside in the tissue are complex and diverse, reflecting the intricate metabolic and immune changes that occur in AT. AT can expand through either adipocyte hyperplasia or hypertrophy, resulting in various effects such as hypoxia, adipocyte cell death, increased secretion of chemokines, and disturbances in fatty acid flow [11].

In 2007, a model called “phenotypic switching” was presented by Saltiel and colleagues to explain how the increased infiltration of ATMs worsens the inflammation associated with obesity. According to their model, obesity causes a shift in macrophage polarization, with the anti-inflammatory “alternatively activated” M2 form, which typically accumulates during negative energy balance, changing into a more pro-inflammatory “classically activated” M1 form. The M1 population is positively associated with IR and is predominant during overeating, as it enhances pro-inflammatory responses, particularly in response to the increase of free fatty acids [12].

Some studies of rodent models with induced obesity showed that macrophage accumulation in white adipose tissue (WAT) was related to IR [6]. Changes in visceral adipose tissue (VAT) can more often lead to obesity-associated pathology than overall adiposity or changes in subcutaneous adipose tissue (SAT) [13]. AT of obese mice shows an increased number of M1 macrophages [14], even higher in VAT than SAT [15,16,17]. M2 macrophages show an upregulated expression of CD163, CD206, Dectin-1, scavenger receptors A and B-1, CCR2, CXCR1, CXCR2, and MgL 1/2 [18].

Collagen is an important component of the extracellular matrix (ECM) in AT representing a significant part of the noncellular mass. Adipocytes are the main producers of collagen, but endothelial cells, preadipocytes, and stem cells can also produce it [19]. Collagens play a critical role in migration, morphogenesis, cell adhesion, differentiation, and wound healing in AT [20]. Collagen IV is an essential element of the basement membrane of adipocytes and is substantial for their survival. Collagen I is the main component of the ECM of AT and makes up most of it, however, in obesity, the accumulation of collagen in AT can lead to fibrosis [21,22]. This leads to the stiffening of the tissue, a decreased ability to expand, and the development of IR. Studies on obese mice fed a HFD have shown that collagens IV, I, III, V, and VI accumulate in AT [23]. ECM plays a critical role in maintaining the structural and cellular integrity of AT, providing mechanical signals for growth and differentiation, and facilitating intercellular communication [24].

In the early stages of adipocyte hypertrophy and adipogenesis, there is increased deposition of ECM components. As the size of the AT changes, the ECM must be extensively reorganized [25]. During hypertrophy, there is an accumulation of ECM components in AT that undergo dynamic changes and their degradation is controlled by extracellular proteolytic enzymes. However, excessive accumulation of ECM components leads to fibrosis, which reduces the mechanical flexibility of the ECM of AT. This, in turn, impairs with adipocyte capacity to expand in response to nutrient demand, resulting in decreased ability to store and bind lipids, and as a result, there is increased deposition of lipids in tissues that cannot store them [26].

The aim of this study was to examine if high-fat diet of mothers or offspring and intergenerational change of diet results in changes of collagen accumulation and macrophage infiltration in subcutaneous and visceral adipose tissue.

## 2. Materials and Methods

### 2.1. Study Protocol

**Figure 1:**
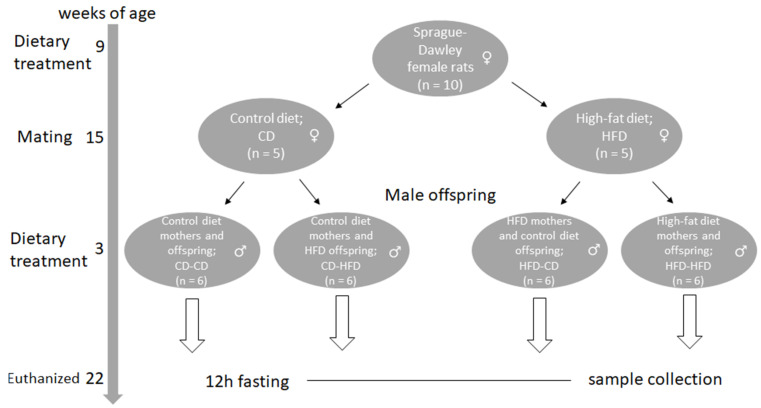
Schematic flowchart of the experiment and duration of control and high-fat diet in mothers and offspring of Sprague Dawley rats.

The study was performed at the Faculty of Medicine University of Osijek and approved by the Croatian Ministry of Agriculture. It involved ten female Sprague Dawley rats randomly divided into two groups and fed either a diet rich in saturated fatty acids (HFD group) or control diet (CD group). The same diets were fed to their male offspring (n = 6 in each group) after birth and during the 3-week lactation period, as previously described [27,28,29]. The flowchart of the experimental procedure is shown in Figure 1. The high-fat diet consisted of 30% wheat, 28% palm oil, 27.2% proteins (soybean), 9.7% forage (corn, soybean, dehydrated alfalfa, animal yeast, corn gluten, animal chalk, salt, monocalcium phosphate, molasses, magnesium oxide), 3.9% vitamins and minerals (vitamin D3, vitamin A, iron, copper, manganese, cobalt, zinc, and iodine) and 0.1% amino acids (lysine, methionine, cysteine, tryptophan, arginine, valine, isoleucine, threonine) (Žito d.o.o., Osijek, Croatia), standard laboratory chow was purchased from Mucedola (Settimo Milanese, Italy). The diet was designed using some previous studies and with help of food technologists [30]. Rats were housed in an air-conditioned room with an equal light and dark cycle and water available ad libitum. The litters were kept the same size to avoid the impact of the number of pups on the results. Animals on a high-fat diet were fed at 9 a.m. and 4 p.m., with an adequate amount of food (30 g) that avoided obesity induced by overfeeding [31].

### 2.2. Sample Collection

Male rat offspring were euthanized at the age of 22 weeks. They were anaesthetized with a combination of ketamine and midazolam (Ketanest S, 25 mg/mL, 3 mL, Midazolam, 5 mg/mL, 1 mL, Pfizer, NY, USA) as described before [29]. Three compartments of WAT were isolated: subcutaneous, epididymal, and perirenal, and immediately stored in plastic tubes with formalin for fixation for a minimum of 12 h. After fixation, tissue samples were embedded in paraffin blocks, and then cut to 6 µm using a microtome (Leica, Vienna, Austria). The sections were stained with Mallory’s trichrome stain, slides were examined, and images were acquired using a digital camera (Olympus, Tokyo, Japan) attached to a microscope (Carl Zeiss Microscopy, White Plains, NY, USA). After being taken, images were saved for further analysis.

### 2.3. Collagen Quantification

Collagen content in ECM was measured using Fiji [32], a distribution of ImageJ software, with a threshold tool and Colour Deconvolution plug-in. Colour Deconvolution is a plug-in developed by Ruifrok et al. in Fiji and used to separate a three-channel image into three colors [33]. Before using the Colour Deconvolution plug-in, input images were converted to RGB images. After that, sections stained with Mallory’s trichrome stain were processed using the Colour Deconvolution plug-in to deconvolve images to their red, green, and blue components; the blue component was identified as collagen content with maximum separation from another ECM content (or tissue). After detecting the best image color component to represent collagen fibers, further analysis was performed using the Threshold tool. The Threshold tool was manually used for every image until the blue area was highlighted in red. After threshold analysis, measurements that were previously set (fraction area, area, limit to threshold) were shown in the results window and then used for further statistical evaluation. This method was modified from that described by Ying Chen et al. [34] for collagen fiber quantification in atherosclerotic lesions.

### 2.4. Immunohistochemistry

Immunohistochemistry for CD68 and CD163 was performed on WAT from each compartment using a fully automated workflow system (Ventana Medical System Inc., AZ, USA). The used primary antibodies were anti-CD68 (KP-1) mouse monoclonal antibody, class IgG1 kappa (Ventana Medical System Inc., Oro Valley, AZ, USA) in the dilution 1:100 and rabbit monoclonal anti-CD163 antibody (ab182422, Abcam, Cambridge, UK) in the same dilution. The number of CD68^+^ and CD163^+^ cells was counted in mm^2^ of tissue using the Olympus CX40 microscope (Olympus, Tokyo, Japan) in sextuplicate, magnification 400×, with a histomorphometric grid representing a surface of 0.17 mm^2^ [35].

### 2.5. Statistical Analysis

Statistical tests were performed using the statistical software SPSS (version 21, IBM Corporation, Armonk, NY, USA), and *p*-values were set as statistically significant at <0.05. Results were checked for distribution normality using the Shapiro–Wilk test. Because of normal distribution, numerical data were expressed as means ± SD, and between-group analysis was performed using ANOVA. For testing of two independent groups the LSD post hoc test was used.

## 3. Results

### 3.1. Collagen Percentage

The collagen deposition in ECM was the lowest in the control group in both SAT and VAT compared to other groups (Table 1).

**Figure 2:**
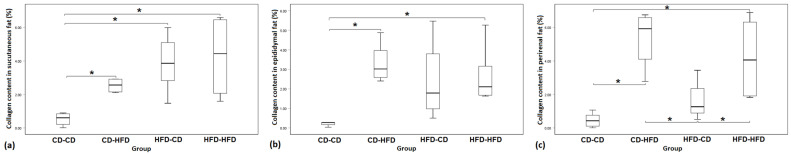
Collagen content (%) in: (**a**) subcutaneous, (**b**) epididymal and (**c**) perirenal adipose tissue; with statistical significance between groups. Data are presented using Whiskers bar graphs (mean, 5–95 percentile). Post hoc LSD test was used to test two independent groups, * *p* < 0.05 was considered significant. CD—standard laboratory chow, HFD—high-fat diet.

**Figure 3:**
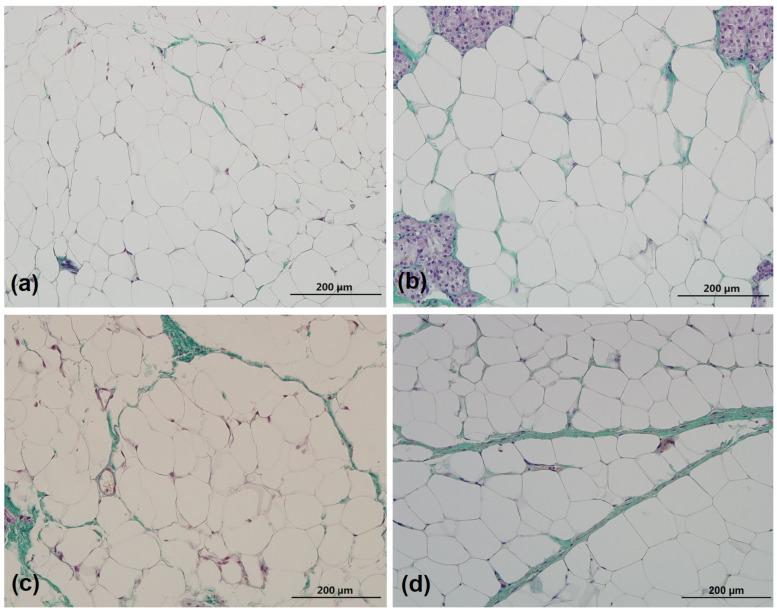
Representative images of collagen staining with Mallory’s trichrome staining in subcutaneous adipose tissue (collagen is visible in green color) of the groups of male rat offspring: (**a**) CD-CD group, (**b**) CD-HFD group, (**c**) HFD-CD group, and (**d**) HFD-HFD group. Magnification: 200×. Scale bar: 200 µm. CD—standard laboratory chow, HFD—high-fat diet.

In SAT, the highest collagen percentage was found in HFD-HFD group, and all groups had a significantly higher collagen percentage than the pups in control group (Figure 2a, representative image of staining in Figure 3). The highest collagen deposition in the epididymal and perirenal depot was found in CD-HFD group followed by HFD-HFD group. That difference is significant compared to control group (Figure 2b,c). The collagen percentage in HFD-CD group was not significantly higher than the collagen content of the control group in epididymal fat, but in perirenal fat, it was lower compared to high fat-fed offspring (groups CD-HFD and HFD-HFD; Figure 2c).

### 3.2. Immunohistochemistry for CD68 and CD163

CD68 was used as a general macrophage marker. It closely correlates to the real number of macrophages in adipose tissue (ATMs). The counted number of CD68^+^ cells per mm^2^ of both SAT and VAT was the highest in HFD-HFD group. This difference was not statistically significant (ANOVA, *p* = 0.485).

**Figure 4:**
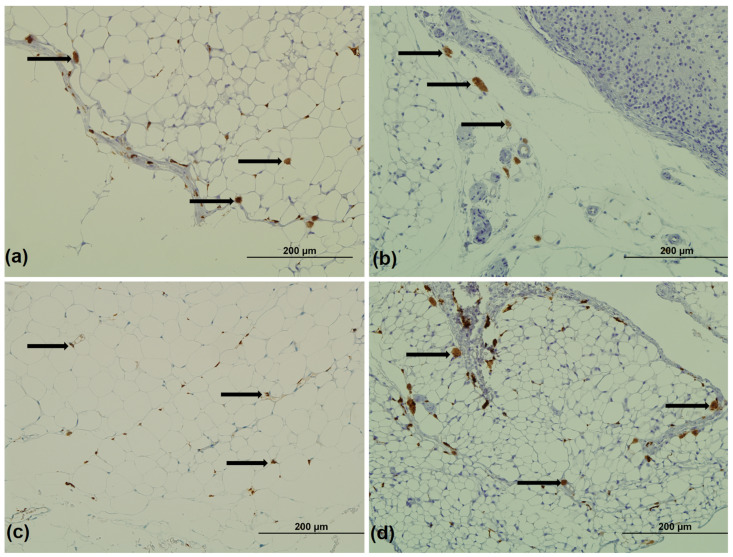
Representative images of CD163^+^ cells (arrow) in perirenal adipose tissue: (**a**) CD-CD group, (**b**) CD-HFD group, (**c**) HFD-CD group, and (**d**) HFD-HFD group of male rat offspring. Magnification: 200×. Scale bar: 200 µm.

After the number of CD68^+^ and CD163^+^ cells per mm^2^ of adipose tissue was counted (representative image of staining in Figure 4), the ratio of CD163 ATMs relative to CD68 was examined.

**Figure 5:**
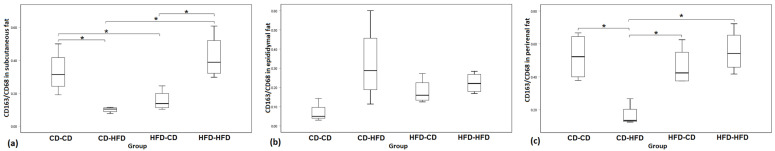
Polarization of macrophage markers among groups of offspring where the number of CD163^+^ cells is presented relative to the number of CD68^+^ cells in: (**a**) subcutaneous, (**b**) epididymal and (**c**) perirenal adipose tissue, with statistical significance comparing groups. Data are presented using Whiskers bar graphs (mean, 5–95 percentile). Post hoc LSD test was used for testing two independent groups, * *p* < 0.05 was considered significant. CD—standard laboratory chow, HFD—high-fat diet.

In SAT, a statistical significance was found when comparing the number of CD163/CD68 cells among groups (ANOVA; *p* = 0.002), and the groups of offspring with unchanged diet had a higher CD163/CD68 number compared to groups with postnatal diet switch (Figure 5a). The highest number of CD163/CD68 cells in epididymal adipose tissue was found in CD-HFD group, but there was no statistical significance among groups (ANOVA; *p* = 0.051; Figure 5b). In comparison, in perirenal adipose tissue, the CD-HFD group had the lowest number of CD163/CD68 cells. This was significantly lower compared to all other groups, as shown in Figure 5c.

## 4. Discussion

The accumulation of collagen in AT is linked to many pathological conditions caused by obesity [36], but ECM multiplication replacing tissue fragments and dead cells is also a physiological regenerative step [37]. Studies show that the lack of collagen VI in HFD-fed or ob/ob mice results in unsuppressed adipocyte hypertrophy, but improved metabolic and inflammatory profile [38]. Obesity represents a state of chronic “silent” inflammation in AT, which could be ATMs contributing to IR [39].

As mentioned before, ECM provides structural support to AT and is composed of a complex network of proteins, glycosaminoglycans, and other components that regulate cell behavior. Studies have shown that changes in ECM can have profound effects on adipocyte differentiation, proliferation, and function. For example, increased ECM stiffness has been shown to promote adipocyte differentiation and enhance lipid accumulation [40].

Conversely, ECM degradation and remodeling by matrix metalloproteinases can lead to adipocyte hypertrophy and inflammation [11]. Furthermore, AT remodeling involves dynamic changes in ECM composition and organization. During adipocyte differentiation and expansion, there is a significant increase in the deposition of collagen and other ECM components [19]. However, in pathological conditions such as obesity, there is a shift towards a more fibrotic ECM phenotype, characterized by increased deposition of collagens and other ECM proteins [41].

In our study, the highest Mallory’s trichrome staining in ECM was shown in the VAT of offspring with changed postnatal diet to HFD. This could be a sign of tissue remodeling with increased collagen deposition, which could potentially be consistent with the tissue reaction to inflammation. In our previous study, we showed that the highest serum concentration of TNF-α was found in offspring with changed postnatal diet [28]. We already know that obesity causes the hypertrophy of adipocytes, production of ECM, and adipocyte apoptosis.

Angiogenesis is a physiological process in which new blood vessels develop from existing vessels and is essential for the maintenance of normal tissue physiology and for tissue remodeling and expansion. AT is one of the most vascularized tissues in the body, and blood vessels supply the tissue with essential nutrients, oxygen, hormones, growth factors, and cytokines [42,43]. Angiogenesis usually occurs before adipogenesis. When WAT expands, active angiogenesis is required to support adipocyte differentiation. However, in certain diseases such as obesity, diabetes, cancer, and cardiovascular disease, angiogenesis may be impaired and play an important role in the progression of these conditions. Although WAT requires new vessels to increase tissue size, the excessive growth of adipose tissue may not be accompanied by a similar increase in angiogenesis, resulting in tissue dysfunction [44]. The rapid expansion of adipocytes can lead to an inadequate blood supply in the surrounding area, causing a localized hypoxic microenvironment in adipose tissue. The presence of hypoxia in AT boosts the expression of hypoxia-inducible factor-1α, which promotes the infiltration of inflammatory cells and contributes to the development of AT fibrosis [11].

In our previous research, the highest adipocyte surface area in subcutaneous and epididymal adipose tissues was also found in groups with altered postnatal diets (CD-HFD and HFD-CD) [29]. Macrophages can enhance the expression of type I collagen, type VI collagen, and matrix metalloproteinases in (pre)adipocytes, meaning that these non-adipose cells and adipocytes could affect the content of ECM [45]. The interaction between macrophages and adipocytes is bidirectional, with macrophages secreting cytokines and other factors that can influence adipocyte function and metabolism.

In addition, macrophages are involved in AT remodeling through their role in the clearance of dead adipocytes and ECM remodeling. In response to adipocyte death, macrophages can engulf and remove the cellular debris, a process known as efferocytosis. This process is essential for the maintenance of tissue homeostasis and can prevent the development of chronic inflammation and fibrosis [46].

However, in obesity, the increased number of adipocytes undergoing programmed cell death can overwhelm the capacity of macrophages to clear the debris, leading to the accumulation of dead cells [6]. There is a known link between obesity and insulin resistance and these cytokines and chemokines play a critical role in the development of obesity and associated metabolic complications by acting as important mediators of macrophage phenotype in adipose tissue and facilitating communication between adipocytes and macrophages [47].

We showed that the number of CD163^+^ cells was higher compared with the number of CD68^+^ cells in SAT in groups of pups with unchanged postnatal diets. CD163, being a staining label for M2 macrophages [48], may indicate a more anti-inflammatory switch in macrophage polarization in offspring that were adapted to the postnatal feeding regime in utero. Studies have shown that HFD-induced obesity leads to an increase in collagen deposition in AT, which is associated with a reduction in the number of CD163^+^ macrophages. This reduction in CD163^+^ macrophages is thought to contribute to the development of chronic low-grade inflammation in AT, which can lead to IR and metabolic dysfunction [38].

Although we did not show any statistical difference among macrophage population in epididymal fat in other compartment of VAT (the perirenal adipose tissue), the lowest number of CD163^+^ cells compared to CD68^+^ cells was found in the group of offspring with the diet changed to HFD (CD-HFD). Keeping in mind that collagen deposition was higher in both groups of HFD-fed offspring (CD-HFD and HFD-HFD) compared to the control group in that AT compartment, but the CD163/CD68 number of ATMs was higher in HFD-HFD pups. This could suggest that adaptation during the prenatal period helped the offspring develop a more favorable anti-inflammatory macrophage polarization. Collagen VI is involved in the regulation of adipocyte differentiation and inflammation, and alterations in collagen expression and the number of CD163^+^ macrophages in AT play a crucial role in the development of obesity-related metabolic dysfunction [38,49]. Khan et al. have discovered a strong link between inflammation in AT and ECM. They observed that the size of adipocytes in ob/ob mice was found to be smaller than adipocytes in collagen VI knockout ob/ob mice. The normalization of blood glucose and other metabolic parameters was observed, indicating that collagen VI, and possibly other components of the ECM, may limit adipocyte expansion during obesity. Additionally, these mice showed a reduction in tissue fibrosis and inflammation in WAT and an increased rate of fatty acid consumption. This suggests that there is a link between AT fibrosis and IR and that inflammatory response plays a crucial role in the remodeling of AT and affects insulin sensitivity [38].

Studies show that genetically modified mice with impaired M2 macrophage activation are likely to suffer from obesity and insulin resistance [50]. Type 2 diabetes mellitus (T2DM) is associated with IR, inflammation, and hypoxia in WAT [51]. In patients with T2DM, hyperglycemia leads to an increase in collagen VI and other ECM accumulation in WAT. Fibrosis of AT can have significant pathophysiological effects on systemic metabolic changes, similar to fibrosis in the kidney, liver, and heart [36]. AT plays a critical role in maintaining a healthy energy balance. It performs important functions such as handling lipids throughout the organism, acting as a primary energy storage site, insulating the body, and secreting various hormones. Any disturbances to these processes are closely related to severe metabolic disorders such as obesity, lipodystrophy, metabolic syndrome, and cachexia [52].

A potential limitation of our study is its sample size. Although many researchers consider six animals per group as an adequate sample size, it may be too small to identify discrete fluctuations in the total macrophage number in adipose tissues. Because only immunohistochemistry was used to identify macrophage polarization, other methods could be considered. Gene expression could also be used in the future to reinforce these results.

## 5. Conclusions

We performed a simple evaluation of collagen content in adipose tissue, and showed that the diets of the mothers and postnatal offspring can lead to changes in collagen deposition and ATM polarization.

The collagen content in SAT was higher in groups where either the mothers or offspring were fed a high-fat diet. In VAT, the high-fat diet in offspring resulted in greater collagen deposition. In SAT, the polarization of ATMs was more affected by the change to a postnatal diet, and in perirenal adipose tissue, the postnatal change to a high-fat diet resulted in a more inflammatory type of macrophage polarization. This could indicate that high-fat diet and especially the postnatal change to a high-fat diet induces a dysfunctional adipose tissue expansion with an increased number of ECM deposits and chronic inflammation, which could affect and disrupt metabolic homeostasis and lead to disease development.

## Figures and Tables

**Table 1:** Collagen percentage in different compartments of white adipose tissue (%).

Group	CD-CD	CD-HFD	HFD-CD	HFD-HFD	*p*-Value
Subcutaneous fat	0.53 ± 0.40	2.84 ± 0.99	3.88 ± 1.76	4.26 ± 2.57	*p* = 0.014
Epididymal fat	0.46 ± 0.61	3.37 ± 1.04	2.39 ± 2.16	2.77 ± 1.53	*p* = 0.027
Perirenal fat	0.47 ± 0.44	5.34 ± 1.80	1.74 ± 1.52	4.18 ± 2.13	*p* = 0.001

Data are presented as mean ± standard deviation. ANOVA; *p* < 0.05. Groups: CD—standard laboratory chow, HFD—high-fat diet.

## Data Availability

Not applicable.

## References

[1] Hotamisligil G.S., Shargill N.S., Spiegelman B.M. (1993). Adipose Expression of Tumor Necrosis Factor-α: Direct Role in Obesity-Linked Insulin Resistance. Science.

[2] Hotamisligil G.S., Arner P., Caro J.F., Atkinson R.L., Spiegelman B.M. (1995). Increased adipose tissue expression of tumor necrosis factor-alpha in human obesity and insulin resistance. J. Clin. Investig..

[3] Armitage J., Jensen R., Taylor P., Poston L. (2004). Exposure to a high fat diet during gestation & weaning results in reduced elasticity & endothelial function as well as altered gene expression & fatty acid content of rat aorta. J. Soc. Gynecol. Investig..

[4] Halberg N., Khan T., Trujillo M.E., Wernstedt-Asterholm I., Attie A.D., Sherwani S., Wang Z.V., Landskroner-Eiger S., Dineen S., Magalang U.J. (2009). Hypoxia-Inducible Factor 1α Induces Fibrosis and Insulin Resistance in White Adipose Tissue. Mol. Cell. Biol..

[5] Ortega Martinez de Victoria E., Xu X., Koska J., Francisco A.M., Scalise M., Ferrante A.W., Krakoff J. (2009). Macrophage content in subcutaneous adipose tissue: Associations with adiposity, age, inflammatory markers, and whole-body insulin action in healthy Pima Indians. Diabetes.

[6] Xu H., Barnes G.T., Yang Q., Tan G., Yang D., Chou C.J., Sole J., Nichols A., Ross J.S., Tartaglia L.A. (2003). Chronic inflammation in fat plays a crucial role in the development of obesity-related insulin resistance. J. Clin. Investig..

[7] Shoelson S.E. (2006). Inflammation and insulin resistance. J. Clin. Investig..

[8] Maury E., Ehala-Aleksejev K., Guiot Y., Detry R., Vandenhooft A., Brichard S.M. (2007). Adipokines oversecreted by omental adipose tissue in human obesity. Am. J. Physiol. Metab..

[9] Steppan C.M., Bailey S.T., Bhat S., Brown E.J., Banerjee R.R., Wright C.M., Patel H.R., Ahima R.S., Lazar M.A. (2001). The hormone resistin links obesity to diabetes. Nature.

[10] Weisberg S.P., McCann D., Desai M., Rosenbaum M., Leibel R.L., Ferrante A.W. (2003). Obesity is associated with macrophage accumulation in adipose tissue. J. Clin. Investig..

[11] Sun K., Kusminski C.M., Scherer P.E. (2011). Adipose tissue remodeling and obesity. J. Clin. Investig..

[12] Lumeng C.N., Bodzin J.L., Saltiel A.R. (2007). Obesity induces a phenotypic switch in adipose tissue macrophage polarization. J. Clin. Investig..

[13] Stolk R.P., Meijer R., Mali W.P., Grobbee D.E., van der Graaf Y., Secondary Manifestations of Arterial Disease Study Group (2003). Ultrasound measurements of intraabdominal fat estimate the metabolic syndrome better than do measurements of waist circumference. Am. J. Clin. Nutr..

[14] Olefsky J.M., Glass C.K. (2010). Macrophages, Inflammation, and Insulin Resistance. Annu. Rev. Physiol..

[15] Strissel K.J., Stancheva Z., Miyoshi H., Perfield J.W., DeFuria J., Jick Z., Greenbreg A.S., Obin M.S. (2007). Adipocyte death, adipose tissue remodeling, and obesity complications. Diabetes.

[16] Harman-Boehm I., Blüher M., Redel H., Sion-Vardy N., Ovadia S., Avinoach E., Shai I., Klöting N., Stumvoll M., Bashan N. (2007). Macrophage Infiltration into Omental Versus Subcutaneous Fat across Different Populations: Effect of Regional Adiposity and the Comorbidities of Obesity. J. Clin. Endocrinol. Metab..

[17] Murano I., Barbatelli G., Parisani V., Latini C., Muzzonigro G., Castellucci M., Cinti S. (2008). Dead adipocytes, detected as crown-like structures, are prevalent in visceral fat depots of genetically obese mice. J. Lipid Res..

[18] Helming L., Gordon S. (2009). Alternative Activation of Macrophages: An Immunologic Functional Perspective. Annu. Rev. Immunol..

[19] Mariman E.C.M., Wang P. (2010). Adipocyte extracellular matrix composition, dynamics and role in obesity. Cell. Mol. Life Sci..

[20] Lin D., Chun T.-H., Kang L. (2016). Adipose extracellular matrix remodelling in obesity and insulin resistance. Biochem. Pharmacol..

[21] Buechler C., Krautbauer S., Eisinger K. (2015). Adipose tissue fibrosis. World J. Diabetes.

[22] Pasarica M., Gowronska-Kozak B., Burk D., Remedios I., Hymel D., Gimble J., Ravussin E., Bray G.A., Smith S.R. (2009). Adipose Tissue Collagen VI in Obesity. J. Clin. Endocrinol. Metab..

[23] Huber J., Löffler M., Bilban M., Reimers M., Kadl A., Todoric J., Zeyda M., Geyeregger R., Schreiner M., Weichhart T. (2006). Prevention of high-fat diet-induced adipose tissue remodeling in obese diabetic mice by n-3 polyunsaturated fatty acids. Int. J. Obes..

[24] Williams A.S., Kang L., Wasserman D.H. (2015). The extracellular matrix and insulin resistance. Trends Endocrinol. Metab..

[25] Nakajima I., Muroya S., Tanabe R.-I., Chikuni K. (2002). Extracellular matrix development during differentiation into adipocytes with a unique increase in type V and VI collagen. Biol. Cell.

[26] Li Q., Hata A., Kosugi C., Kataoka N., Funaki M. (2010). The density of extracellular matrix proteins regulates inflammation and insulin signaling in adipocytes. FEBS Lett..

[27] Perić Kačarević Ž., Grgić A., Šnajder D., Bijelić N., Belovari T., Cvijanović O., Blažičević V., Radić R. (2017). Different combinations of maternal and postnatal diet are reflected in changes of hepatic parenchyma and hepatic TNF-alpha expression in male rat offspring. Acta Histochem..

[28] Peric Kacarevic Z., Snajder D., Maric A., Bijelic N., Cvijanovic O., Domitrovic R., Radic R. (2016). High-fat diet induced changes in lumbar vertebra of the male rat offsprings. Acta Histochem..

[29] Najder D., Perić Kačarević Ž., Grgić A., Bijelić N., Fenrich M., Belovari T., Radić R. (2019). Effect of different combination of maternal and postnatal diet on adipose tissue morphology in male rat offspring. J. Matern. Neonatal. Med..

[30] Frihauf J.B., Fekete M., Nagy T.R., Levin B.E., Zorrilla E.P. (2016). Maternal Western diet increases adiposity even in male offspring of obesity-resistant rat dams: Early endocrine risk markers. Am. J. Physiol. Integr. Comp. Physiol..

[31] Laaksonen S., Nevalainen T.O., Haasio K., Kasanen I.H.E., Nieminen P., Voipio H.-M. (2013). Food and water intake, growth, and adiposity of Sprague-Dawley rats with diet board for 24 months. Lab. Anim..

[32] Schindelin J., Arganda-Carreras I., Frise E., Kaynig V., Longair M., Pietzsch T., Preibisch S., Rueden C., Saalfeld S., Schmid B. (2012). Fiji: An open-source platform for biological-image analysis. Nat. Methods.

[33] Ruifrok A.C., Johnston D.A. (2001). Quantification of histochemical staining by color deconvolution. Anal. Quant. Cytol. Histol..

[34] Chen Y., Yu Q., Xu C.-B. (2017). A convenient method for quantifying collagen fibers in atherosclerotic lesions by ImageJ software. Int. J. Clin. Exp. Med..

[35] Van Steenberghe M., Schubert T., Guiot Y., Bouzin C., Bollen X., Gianello P. (2017). Enhanced vascular biocompatibility of decellularized xeno-/allogeneic matrices in a rodent model. Cell Tissue Bank..

[36] Sun K., Tordjman J., Clément K., Scherer P.E. (2013). Fibrosis and Adipose Tissue Dysfunction. Cell Metab..

[37] Lupher M., Thannickal V.J., Wynn T.A. (2013). Host Responses in Tissue Repair and Fibrosis. Annu. Rev. Pathol. Mech. Dis..

[38] Khan T., Muise E.S., Iyengar P., Wang Z.V., Chandalia M., Abate N., Zhang B.B., Bonaldo P., Chua S., Scherer P.E. (2009). Metabolic Dysregulation and Adipose Tissue Fibrosis: Role of Collagen VI. Mol. Cell. Biol..

[39] Neels J.G., Olefsky J.M. (2005). Inflamed fat: What starts the fire?. J. Clin. Investig..

[40] Baker R.G., Hayden M.S., Ghosh S. (2011). NF-κB, inflammation, and metabolic disease. Cell Metab..

[41] Divoux A., Tordjman J., Lacasa D., Veyrie N., Hugol D., Aissat A., Basdevant A., Guerre-Millo M., Poitou C., Zucker J.-D. (2010). Fibrosis in Human Adipose Tissue: Composition, Distribution, and Link With Lipid Metabolism and Fat Mass Loss. Diabetes.

[42] Lemoine A.Y., LeDoux S., Larger E. (2013). Adipose tissue angiogenesis in obesity. Thromb. Haemost..

[43] Cao Y. (2007). Angiogenesis modulates adipogenesis and obesity. J. Clin. Investig..

[44] Carmeliet P., Jain R.K. (2011). Molecular mechanisms and clinical applications of angiogenesis. Nature.

[45] Mori S., Kiuchi S., Ouchi A., Hase T., Murase T. (2014). Characteristic Expression of Extracellular Matrix in Subcutaneous Adipose Tissue Development and Adipogenesis; Comparison with Visceral Adipose Tissue. Int. J. Biol. Sci..

[46] Hotamisligil G.S. (2006). Inflammation and metabolic disorders. Nature.

[47] Cai Z., Huang Y., He B. (2022). New Insights into Adipose Tissue Macrophages in Obesity and Insulin Resistance. Cells.

[48] Aron-Wisnewsky J., Tordjman J., Poitou C., Darakhshan F., Hugol D., Basdevant A., Aissat A., Guerre-Millo M., Clément K. (2009). Human Adipose Tissue Macrophages: M1 and M2 Cell Surface Markers in Subcutaneous and Omental Depots and after Weight Loss. J. Clin. Endocrinol. Metab..

[49] Wentworth J.M., Naselli G., Brown W.A., Doyle L., Phipson B., Smyth G.K., Wabitsch M., O’Brien P.E., Harrison L.C. (2010). Pro-Inflammatory CD11c+CD206+ Adipose Tissue Macrophages Are Associated With Insulin Resistance in Human Obesity. Diabetes.

[50] Odegaard J.I., Ricardo-Gonzalez R.R., Goforth M.H., Morel C.R., Subramanian V., Mukundan L., Red Eagle A., Vats D., Brombacher F., Ferrante A.W. (2007). Macrophage-specific PPARgamma; controls alternative activation and improves insulin resistance. Nature.

[51] Ohno H., Matsuzaka T., Tang N., Sharma R., Motomura K., Shimura T., Satoh A., Han S.-I., Takeuchi Y., Aita Y. (2018). Transgenic Mice Overexpressing SREBP-1a in Male ob/ob Mice Exhibit Lipodystrophy and Exacerbate Insulin Resistance. Endocrinology.

[52] Vegiopoulos A., Rohm M., Herzig S. (2017). Adipose tissue: Between the extremes. EMBO J..

